# Neural networks subtract and conquer

**DOI:** 10.7554/eLife.26157

**Published:** 2017-04-26

**Authors:** Guillaume Hennequin

**Affiliations:** 1 Computational and Biological Learning Laboratory, Department of Engineering, University of Cambridge, Cambridge, United Kingdomg.hennequin@eng.cam.ac.uk

**Keywords:** recurrent neural networks, learning, cognition, modeling, working memory, decision making, None

## Abstract

Two theoretical studies reveal how networks of neurons may behave during reward-based learning.

**Related research articles** Miconi T. 2017. Biologically plausible learning in recurrent neural networks reproduces neural dynamics observed during cognitive tasks. *eLife* **6**:e20899. doi: 10.7554/eLife.20899

Song HF, Yang GR, Wang XJ. 2017. Reward-based training of recurrent neural networks for cognitive and value-based tasks. *eLife* **6**:e21492. doi: 10.7554/eLife.21492

To thrive in their environments, animals must learn how to process lots of inputs and take appropriate actions (Figure 1A). This sort of learning is thought to involve changes in the ability of synapses (the junctions between neurons) to transmit signals, with these changes being facilitated by rewards such as food. However, reward-based learning is difficult because reward signals do not provide specific instructions for individual synapses on how they should change. Moreover, while the latest algorithms for reinforcement learning achieve human-level performance on many problems (see, for example, Mnih et al., 2015), we still do not fully understand how brains learn from rewards. Now, in eLife, two independent theoretical studies shed new light on the neural mechanisms of learning.

The studies address two complementary aspects of reward-based learning in recurrent neuronal networks – artificial networks of neurons that exhibit dynamic, temporally-varying activity. In both studies, actions are generated by a recurrent network (the “decision network”) that is composed of hundreds of interconnected neurons that continuously influence each others’ activity (Figure 1). The decision network integrates sensory information about the state of the environment and responds with an action that may or may not result in a reward. The network can also change the ability of individual synapses to transmit signals, referred to as synapse strength. Over a period of time, increasing the strength of synapses that promote an action associated with a reward leads to the network choosing actions that receive rewards more often, which results in learning.

**Figure 1.**
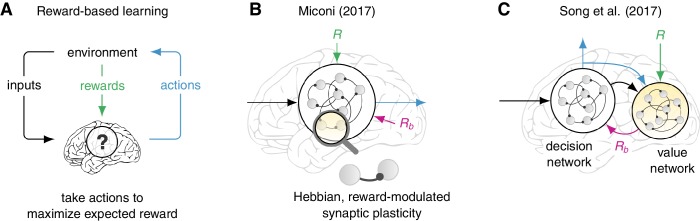
Models for reward-based learning in neural networks. (**A**) Many behavioral tasks can be formulated as reward-based (or reinforcement) learning problems: the animal learns to use sensory inputs to perform actions that maximize the expected reward. Miconi and, independently, Song et al. addressed two complementary aspects of how brain circuits might solve such problems. (**B**) Miconi studied a biologically plausible form of a synaptic plasticity rule (ability of a synapse to strengthen or weaken), which is modulated by reward and is capable of learning complex tasks by adjusting the connectivity of a “decision network” (Miconi, 2017). The strengths of synapses are modified according to a function of the electrical activities on each side of the synapse and a delayed reward signal (R) delivered at the end of each trial. Critically, successful learning requires an appropriate reward baseline (R_b_) to be subtracted from the actual reward, but exactly how this baseline can be estimated by another circuit is not addressed. (**C**) Song et al. show that the total future reward can be estimated dynamically by a separate “value network” that integrates the rewards received from the environment as well as the activity (and outputs) of the decision network (Song et al., 2017). The output of the value network then serves as a reward baseline used to modulate a mathematically optimal, but biologically infeasible, rule that governs the synaptic plasticity in the decision network. Neurons are shown as gray circles, and synapses as black lines with a circle at one end.

At the core of both studies lies a classic algorithm for reinforcement learning known as REINFORCE, which aims to maximize the expected reward in such scenarios (Figure 1A; Williams, 1992). In this algorithm, the strength of a synapse that connects neuron j to neuron i, W_ij_, changes to W_ij_ + αE_ij_(t) x (R(t) − R_b_), where α is a constant, E_ij_ is a quantity called the eligibility, t is time, R is the reward and R_b_ is a quantity called the reward baseline. The eligibility E_ij_(t) expresses how much a small change of W_ij_ affects the action taken by the decision network at time t.

The conceptual simplicity of REINFORCE and the fact that it can be applied to the tasks commonly studied in neuroscience labs make it an attractive starting point to study the neural mechanisms of reward-based learning. Yet, this algorithm raises two fundamental questions. Firstly, how can a synapse estimate its own eligibility, using only locally-available information? Indeed, in a recurrent network, a change in synapse strength can influence a third neuron, implying that the eligibility depends on the activity of that third neuron, which the synapse will have never seen. Perhaps more importantly, in scenarios where the reward arrives after the network has produced long sequences of actions, the synapse must search the stream of recently experienced electrical signals for those that significantly influenced the action choice, so that the corresponding synapses can be reinforced. Secondly, how can the network com- pute an adequate reward baseline R_b_?

In one of the papers Thomas Miconi of the Neurosciences Institute in La Jolla reports, somewhat surprisingly, that simply accumulating over time a superlinear function (such as f(x) = x^3^) of the product of the electrical signals on both sides of the synapse, returns a substitute for the optimal synapse eligibility that works well in practice (Miconi, 2017). This form of eligibility turns REINFORCE into a rule for the ability of synapses to strengthen or weaken (a property known as synaptic plasticity) that is more biologically realistic than the original optimal REINFORCE algorithm (Figure 1B) and is similar in spirit to models of synaptic plasticity involving neuromodulators such as dopamine or acetylcholine (Frémaux and Gerstner, 2016).

Miconi’s practical use of a superlinear function seems key to successful learning in the presence of delayed rewards. This nonlinearity tends to discard small (and likely inconsequential) co-fluctuations in electrical activity on both sides of the synapse, while amplifying the larger ones. While a full understanding of the success of this rule will require more analysis, Miconi convincingly demonstrates successful training of recurrent networks on a variety of tasks known to rely on complex internal dynamics. Learning also promotes the emergence of collective dynamics similar to those observed in real neural circuits (for example, Stokes et al., 2013; Mante et al., 2013).

As predicted by the theory of REINFORCE (Peters and Schaal, 2008), Miconi found it essential to subtract a baseline reward (R_b_) from the actual reward (R) obtained at the end of the trial. While Miconi simply assumes that such predictions are available, Francis Song, Guangyu Yang and Xiao-Jing Wang of New York University and NYU Shanghai wondered how the brain could explicitly learn such detailed, dynamic reward predictions (Song et al., 2017). Alongside the main decision network, they trained a second recurrent network, called the “value network”, to continuously predict the total future reward on the basis of past activity in the decision network (including past actions; Figure 1C). These reward predictions were then subtracted from the true reward to guide learning in the decision network. Song et al. were also able to train networks on an impressive array of diverse cognitive tasks, and found compelling similarities between the dynamics of their decision networks and neural recordings.

Importantly, although Song et al. used synapse eligibilities (with a few other machine learning tricks) that are not biologically plausible to train both networks optimally, their setup now makes it possible to ask other questions related to how neurons represent uncertainty and value. For example, when it is only possible to observe part of the surrounding environment, optimal behavior often requires individuals to take their own internal uncertainty about the state of the world into account (e.g. allowing an animal to opt for lower, but more certain rewards). Networks trained in such contexts are indeed found to select actions on the basis of an internal sense of uncertainty on each trial. Song et al. tested their model in a simple economic decision-making task where in each trial the network is offered a choice of two alternatives carrying different amounts of rewards. They found that there are neurons in the value network that exhibit selectivity to offer value, choice and value, or choice alone. This is in agreement with recordings from the brains of monkeys performing the same task.

The complementary findings of these two studies could be combined into a unified model of reward-based learning in recurrent networks. To be able to build networks that not only behave, but also learn, like animals promises to bring us closer to understanding the neural basis of behavior. However, progress from there will rely critically on our ability to analyze the time-dependent strategies used by trained networks (Sussillo and Barak, 2013), and to identify neural signatures of such strategies.
